# Nanotopography in directing osteogenic differentiation of mesenchymal stem cells: potency and future perspective

**DOI:** 10.2144/fsoa-2021-0097

**Published:** 2021-11-18

**Authors:** Anggraini Barlian, Katherine Vanya

**Affiliations:** 1 School of Life Science & Technology, Institute of Technology Bandung, Bandung, West Java, 40132, Indonesia; 2 Research Center for Nanosciences & Nanotechnology, Institute of Technology Bandung, Bandung, West Java, 40132, Indonesia

**Keywords:** nanopattern, osteogenic, stem cells, tissue engineering

## Abstract

Severe bone injuries can result in disabilities and thus affect a person's quality of life. Mesenchymal stem cells (MSCs) can be an alternative for bone healing by growing them on nanopatterned substrates that provide mechanical signals for differentiation. This review aims to highlight the role of nanopatterns in directing or inducing MSC osteogenic differentiation, especially in bone tissue engineering. Nanopatterns can upregulate the expression of osteogenic markers, which indicates a faster differentiation process. Combined with growth factors, nanopatterns can further upregulate osteogenic markers, but with fewer growth factors needed, thereby reducing the risks and costs involved. Nanopatterns can be applied in scaffolds for tissue engineering for their lasting effects, even *in vivo*, thus having great potential for future bone treatment.

Bone fracture is one of the causes of disabilities that affect a person's quality of life. Bone tissue has the ability to heal by itself, but in some cases the healing process needs help from implants or scaffolds [1]. However, these methods may pose some risks, such as infection, nerve damage or even rejection by the immune system [2]. Therefore, more effective and less risky alternative methods are needed. Mesenchymal stem cells that can cause minimal rejection responses is one such alternative.

Stem cells are a very rapidly developing topic in the biomedical field, especially related to their use in bio-engineering of various tissues [3] such as vascular [4], neuronal [5], bone [6], skin [7] and brain [8] tissues. Stem cells have the characteristics needed in tissue engineering: their abilities, first, to regenerate, and second, to differentiate into different types of cells [9]. Mesenchymal stem cells (MSCs), in particular, are very widely used in regenerative medicine because they are multipotent, easier to obtain because they originate from many tissues in the body and have immunomodulating abilities [10]. The role of stem cells as a source of cells in tissue engineering applications is important, including bone tissue engineering.

Regulation of differentiation, proliferation and regeneration of stem cells can occur intracellularly or extracellularly (microenvironment/niche) in the form of the extracellular matrix (ECM), or neighboring cells. The ECM is an important aspect because the cell must adhere to the ECM and receive signals from it [11]. The ECM can not only provide biophysical signals but also provide biochemical signals for example through ECM composition for guiding MSC's osteogenic differentiation [12,13]. Biophysical signals from ECM to cells includes substrate topography [14], matrix stiffness [15], mechanical forces [16] and also matrix viscoelasticity, which has recently been paid much attention [17]. This mechanism is called mechanotransduction, which is a conversion mechanism from mechanical signals into biochemical signals [18]. The rate of cell proliferation and attachment is influenced by the size of the microenvironment and the layout of the other cells within it. Differentiation and cell signaling pathways are also influenced by the biochemical and mechanical composition of the existing microenvironment and, in turn, affect gene expression that occurs in cells [19]. How biophysical cues can direct the fate of the cell in tissue engineering needs consideration in future applications.

Besides the microenvironment, another aspect that influences stem cell development is the epigenetic aspect. Epigenetic factors are intracellular processes that do not involve genetic factors. Epigenetic mechanisms usually cause changes in gene expression without changing the DNA structure or sequence. Some examples of epigenetic mechanisms include cytosine DNA methylation, histone modification by histone acetylation and methylation, and gene expression regulatory activity of small noncoding RNA [20]. Mechanical forces can be transduced into the cell through the cytoskeleton to the chromatin in the nucleus, thereby influencing epigenetic regulation. Nuclear lamins can interact with DNA, chromatin, and histones in the lamina-associated domains (LADs). LADs play a role in the regulation of histone modifications and also changes in chromatin structure through nucleoskeletal reorganization. In LADs, there are heterochromatin markers such as H3K9me3 and H3K27me3 that play a role in stem cell differentiation to prevent cell reprogramming and silencing certain line-specific genes with the outcome that differentiated cells can maintain their identity. However, during cell reprogramming, H3K9me3 functions to maintain the undifferentiated state by suppressing the activation of transcription factors and arranging chromatin into dense heterochromatin, while H3K27me3 plays a role in facultative heterochromatin silencing. Thus, cytoskeleton and lamins rearrangements can regulate histone modifications and thereby influence MSC differentiation. This rearrangement of the cytoskeleton and lamins can occur as a result of mechanical signals by topography [21]. Therefore, substrate topography, in which the cells grow, plays an important role in the onset, which then determines the next step of cell fate.

Stem cells can be administered directly through injection or by transplantation through a scaffold medium [22]. Scaffolds can serve as a place for cells to grow and differentiate *in vitro* in such a way that afterward they can be transplanted into the body. *In vitro* differentiation in tissue culture is usually regulated by adding growth factors to the growth medium. However, the use of growth factors has some limitations, especially *in vivo*. Proteolytic activity or protein degradation by enzymes in the body causes growth factors to become unstable and degrade; as a result, they have to be administered several times or in larger doses than normally found in the body to maintain an effective concentration. Therefore, costs are higher and unwanted effects may also occur [23]. Developments in topographic engineering methods to control cell behavior and differentiation can be a solution to these problems and can increase our understanding of the interactions and signaling processes that occur during MSCs' regulation of differentiation and regeneration [3]. Wang *et al.* were one of the first who showed that topography can affect MSC's differentiation. In their work, they found that elongated topographies can result in neural differentiation rather than osteogenic differentiation [24]. Besides neural proliferation, elongated topographies such as nanogrids was also suitable for diffraction [25].

Micropatterning is a method to engineer substrate topographies that resemble the microenvironment suitable for cells. The substrate is made with micron-sized patterns that define the adhesion of cells to the substrate [19]. Since its discovery, microtopography of substrates has been extensively studied to see how these features interact with cells. However, microenvironments also have nanoscale features. Nanopatterning, or the manufacture of nano motifs on substrates, can now be accomplished due to ever-developing nanotechnology [16]. How far or big the role of nanotechnology in creating microenvironments will greatly affect substrate-dependent osteogenic differentiation.

Nanopattern technology also has many challenges, such as difficulties in manufacturing and designing nanoscale geometries, as well as high costs with relatively low yields [26]. The effect of nanopatterns on osteogenic differentiation is evident through the upregulation of osteogenic markers on nanopattern surfaces compared with controls. Research by Kim *et al.* showed that the expression of Col I, *RUNX2*, and OPN was higher on nanopattern surfaces. In addition, their research also shows that nanopatterns can be combined with biochemical compounds to further improve results [27]. Further, Amaral *et al.* proved that nanopatterns can be embedded into scaffolds and can induce osteogenic differentiation even without osteogenic-inducing medium [28]. The role of nanopatterns in directing osteogenic differentiation is intriguing and also important to understand in the endeavor to develop bone tissue engineering applications.

Nanopatterns have great potential in tissue engineering because they can induce MSC differentiation in scaffolds through biophysical signals, which have several advantages over biochemical compounds such as growth factors. However, fabricating nanopatterns is quite difficult. Moreover, there are many types of nanopatterns, and pattern selection can affect cell contractility, which ultimately affects cell differentiation. Many aspects need to be considered, such as the required technology, the use of materials and the production costs [29]. These factors can be a challenge in the development of nanopattern fabrication methods. This concise review highlights several uses of nanopattern methods in inducing osteogenic differentiation, as well as the potential for their development in the future.

## MSC behavior in topography

MSC behaviors such as migration, adhesion, proliferation and differentiation can be regulated by biophysical signals from the microenvironment/niche [16,30]. This mechanism is called mechanotransduction, which is a conversion mechanism from mechanical into biochemical signals [18]. Mechanotransduction is caused by changes in the cytoskeleton that interacts with the nucleus via the nucleoskeletal lamin [31]. The main receptors in mechanotransduction are integrins. Integrins will mediate cell adhesion to the substrate and form a focal adhesion (FA) complex [32]. FA connects the substrate with cytoskeletal actin filaments (F-actin) [33]. Stress and cytoskeletal remodeling can affect the morphology of the nucleus so that it also has an impact on cell function and growth [16]. Therefore, different engineered patterns from the substrate will cause different cytoskeletal remodeling and in the end, will direct different cell functions. Microenvironments provide both biochemical and biophysical cues which the cells will integrate with their internal regulatory mechanism in order to meet their physiological needs [34]. This phenomenon can be adopted in topography engineering to guide cell differentiation.

Integrin transmembrane receptor consists of α dan β subunits and binds to the RGD and GFOGER ligand motifs found in the ECM's collagen. Integrin-ligand binding causes clustering of cytoplasmic proteins that regulate signaling cascades for several cellular processes such as proliferation and differentiation [16]. The role of integrins in cell-matrix attachment occurs in three mechanical processes. First, the integrin-matrix binding force must pass a certain threshold to be able to withstand the force exerted during adhesion. Second, integrins must link the cytoskeleton with the ECM in order for signals to be transduced into the cell. Finally, biophysical signals need to be translated into biochemical signals through mechanotransduction [33]. It is this mechanism that underlies how nanotopography can play a role in directing MSC's osteogenic differentiation.

When the scaffold is implanted into the body, plasma proteins are adsorbed to the surface of the scaffold in such a manner that its topography and biochemical characteristics will affect the conformation and amount of proteins adsorbed to the scaffold [16]. Cell adhesion to the matrix will cause clustering of integrins which will increase the recruitment process of intracellular proteins that form FA complexes [32,35]. Talin is one of the intracellular proteins that play a role in the formation of FA, and it binds to ß integrin and actin [35]. This binding between talin and integrin plays a role in stabilizing clustering processes and also mediates crosslinking between integrins and F-actin and between proteins attached to F-actin such as vinculin and α-actinin. Vinculin plays a role in stabilizing stress-induced attachment [18]. Vinculin and paxillin share both function and binding sites. However, paxillin does not bind directly to actin as is the case with vinculin [35]. Mature focal adhesion (FA) will usually have these three proteins, namely talin, vinculin and paxillin. Integrin signaling pathways are mediated by a tyrosine kinase called focal adhesion kinase (FAK). FAK plays a role in attachment and also acts as a scaffold for other FA components, and it initiates several signaling cascades [33]. These signaling cascades play a very important role in determining cell fate.

Mechanotransduction can occur directly or indirectly. Indirect mechanotransduction occurs through a series of biochemical signaling from FA formation. Meanwhile, direct mechanotransduction occurs due to conformational changes in the cytoskeleton that connects ECM to the nucleus via nucleoskeleton lamins [31]. Cytoskeletons play a role in regulating various aspects of the cell such as cell shape, migration and also mechanical signal transmission and transduction. Cytoskeleton components consist of F-actin and myosin [36]. Therefore, topography engineering can determine mechanotransduction processes including signaling cascade.

Cytoskeleton stress can affect cell differentiation. Cells cultured on surfaces that allow for high cytoskeleton stress will have greater FA than cells with looser cytoskeletons. High cytoskeleton stress plays an important role in osteogenesis. Therefore, to direct MSC's osteogenic differentiation, nanopatterns must be designed to produce high cytoskeleton stress [31]. Intracellular tension causes deformation of the nuclear membrane, resulting in openings in the nucleopore, which increases mRNA transport and protein translation [16]. This intracellular tension can also affect lamin structures. Lamin itself is a nucleoskeletal intermediate filament that plays a role in providing structure for the nucleus. It is also associated with chromosomes and thus has a role in DNA replication. Lamin is linked to cytoskeletal proteins via the linker of the nucleoskeleton and cytoskeleton (LINC) complex. Changes in lamin organization can affect signaling pathways and compartmentalization in the nucleus such that the position of nuclear components such as chromosomes may also change. This cellular remodeling provides stimulatory effects on lamins and chromosomes with the result that it may also impact gene expression [31].

Topography induces many signaling pathways, one of the most studied of which is the YAP/TAZ pathway. These two proteins are transcriptional coactivators that are homologous to each other. These proteins can translocate from the cytosol to the nucleus when activated by mechanical signals. A stiffer surface will increase integrin clustering and FA formation such that F-actin polymerization and stress fiber formation increase. Cells will spread out more and cover a larger area which will support YAP/TAZ translocation to the nucleus [37]. It is this balance between the ration of YAP and TAZ in the cytoplasm and nucleus that plays a role in the regulation of cell differentiation [37–39]. The role of YAP protein itself in osteogenesis has yet to be determined because contradictory results are often obtained. However, TAZ plays a role in osteogenesis by coactivating transcription by *RUNX2* for osteogenesis as well as inhibiting transcription by *PPARγ* for adipogenesis [37].

Another signaling pathway that plays a particular role in osteoblast maturation is the Wnt/β-catenin pathway. Wnt proteins interact with frizzled (FZD) surface receptors such that the effector β-catenin is translocated to the nucleus while at the same time inhibiting the complex that degrades β-catenin, namely *AXIN1* [40,41]. The Wnt/β-catenin signaling pathway is associated with calcification and osteogenic marker expression. It is also involved in a mutually reinforcing crosstalk with integrins during differentiation. Integrins regulate differentiation via the Wnt pathway, and Wnt increases integrin expression, resulting in further regulation of osteogenic differentiation [40,42]. The signaling pathways mentioned are involved in promoting osteogenic differentiation. Hence, the activity of proteins involved in these pathways, such as YAP and Wnt, can become an indicator or marker of osteogenic differentiation activity in cells [39,42]. Likewise, in nanotopographic engineering, markers from these signaling pathways are used to determine the role of nanopatterns in osteogenesis.

## Development of nanopattern fabrication method

Nanopattern is a substrate engineering method to produce nanoscale structures. The first cultured cells utilizing topographic signals were reported in 1914 [43]. Developments in nanoscale fabrication technology made it possible to create nanotopographic structures. Nanopatterns themselves are used not only in the biological field but also in fields, for instance in electronics for electrochemical cells [44]. Nanopattern developments in the study of cell behavior started about three decades ago [45]. Even now, research on nanopatterns to find the optimal method or combination to direct cells according to needs is ongoing. Developments in this area are, of course, very dependent on advancements in the field of nanotechnology.

Different methods have been used to produce nanopatterns, with the research is still underway because many challenges in nanofabrication remain, such as difficulties in designing and producing nanoscale geometries as well as high costs with low yields [26]. Nanofabrication methods continue to shift over time. Various methods have been carried out through research to produce nanopatterns, such as sonication [46], sandblasting [47], solvothermal method [48], plasma oxidation [49] and other methods. However, several methods that are found quite often are lithography, etching and anodization. Lithography and its variations were used quite widely in the early 2010s [27,50,51], were continued with etching [52,53], and then were shifted to anodization during the end of the decade [54–65]. Developments of various nanopattern fabrication methods, the resulting patterns and the materials used can be seen in Table 1.

**Table 1.** Various nanopattern fabrication methods used for osteogenic differentiation (2010–2021).

Materials	Patterns used	Methods	Ref.
Ti	Nanopores (10 nm diameter) on nano-dimples (120 nm)	Electrochemical nanopattern formation (anodization, sonication, chemical etching)	[64]
Ti	Mesoporous nanostructured coatings	Sonication	[46]
Ti	Grids (micron, nano, micron/nano [hybrid])	Femtosecond laser irradiation	[66]
Ti	Hemisphere-like nanostructures (approx. 50, 100 and 200 nm)	Colloidal lithography of glass substrate and sputter-coating of Ti	[51]
Ti	Nanostructured surface, microstructured surface, untreated surface	Treatment with either HNO_3_-H_2_SO_4_-HCl to produce MS, and H_2_SO_4_-H_2_O_2_ to produce NS	[67]
Ti	Roughness (Ti coated with GO nanomaterial)	Sandblasting and acid etching and ultrasonic atomization spraying technique	[47]
Ti	Nanotubes with modified Tanfloc (TA)-based polyelectrolyte multilayers using heparin and hyaluronic acid	Anodization and layer-by-layer deposition	[65]
Ti	ATi nanotubes coated with GO (roughness)	Anodization and anodic-electrophoretic deposition	[62]
Ti	Nanotubes (30–40 nm), nanograins (60–100 nm) with nanopore structure	Anodization, etching, & anodization after etching	[63]
Ti	TiO_2_ Nanotubes (20, 50, 100 nm)	Anodization	[54]
Ti	Nanotubes (potential: diameter: 30 V: 74 nm; 40 V: 92 nm; 50 V: 112 nm; 60 V: 128 nm; 70 V: 148 nm)	Anodization	[55]
Ti	Nanotubes (flat Ti, amorphous, and anatase crystallinity)	Anodization	[56]
Ti	Nanotubes (average diameters ∼20 (NT1), ∼50 (NT2), & ∼90 nm (NT3), and two-tiered HC surface composed of smaller nanotubes (s-HC) clustered within larger domains (L-HC))	Anodization	[57]
Ti	Nanotubes loaded with strontium (Sr) and lanthanum (La) (nanotubes (TN), Sr-containing TiO2 nanotube (STN), La-loaded SNT (LSTN))	Anodization	[58]
Ti_6_Al_4_V	Nanotubes (ground Ti_6_Al_4_V (Ti), nanostructured Ti_6_Al_4_V (N), Ti_6_Al_4_V incubated in SBF (TiH), nanostructured Ti_6_Al_4_V incubated in SBF (NH)	Anodization	[59]
Ti_6_Al_4_V	Nanotubes (39 & 83 nm)	Anodization	[60]
Ti_6_Al_4_V	Nanostructured biogenic apatite coatings (thickness 450 ± 20 nm)	Ionized jet deposition technology	[68]
TiNbTa	Nanotubes (potential - diameter: 10 V - 18 nm, 20 V - 36 nm, 30 V - 46 nm)	Anodization	[61]
TiCaP	Nanograins from coating (Ti thin film on glass substrate (Ti), CaP thin film on glass substrate (CaP), CaP thin film on glass substrate with Ti interlayer (TiCaP)	Radio frequency magnetron sputter deposition	[69]
Barium titanate (BT) NP/alginate	Porous nanocomposite scaffold	Dispersion of BT NP in water and adding alginate	[28]
HAP/Ti_3_C_2_T_x_	Nanocomposite membrane (ultralong HAP nanowires (UHAPNWs)/MXene (Ti_3_C_2_T_x_) film)	Solvothermal method and mixing and vacuum drying	[48]
PCL	Multi-walled carbon nanotubes (MWCNTs) and nano-hydroxyapatite (nHA) in 3D-printed porous scaffold (PCL/MWCNT, PCL/HA, PCL/HA/MWCNT)	Screw-assisted extrusion-based additive manufacturing system and melt blending method	[70]
PCL	Nanoparticles on nanofibers (PCL, PCL-ZnO, PCL-C-ZnO)	Nanoparticles by carbonization and oxidation, PCL fibers by electrospinning	[71]
HApN/PCL	Nanoparticle hybrid	Emulsification-solvent evaporation technique. HAPN (hydroxyapatite nanoparticle) were prepared using simple wet chemical precipitation technique	[72]
PCL/F-MWCNT	Composite nanofiber PCL/F-MWCNT (PCL nanofibers and functionalized multiwall carbon nanotubes concentration of 0.5, 1, 2 & 3%)	Electrospinning	[73]
PUA	Nanogrooves (width 150 nm, periodicity 300 nm; & width 300 nm, periodicity 600 nm) and nanodots (hexagonal and square; diameter 460 nm, periodicity 600 nm)	Nanopatterned Si-master by photolithography and coating by initiated chemical vapor deposition technique	[27]
PUA	Nanodots (150, 400, 600 nm diameter) and nanolines (150, 400, 600 nm width)	Self-replication and UV-assisted capillary force lithography	[50]
PDMS	Wrinkled topography gradient (amplitude: 144–2854 nm; wavelength: 0.91–13.62 μm)	Shielded plasma oxidation and imprinting lithography	[49]
PDMS	Nanoparticle composite (AuNM/PDMS, SPION/PDMS, GO/PDMS, GQD/PDMS, pristine PDMS)	Immersion of PDMS in 6 types of nanoparticles	[74]
PS	Nanopits (average diameters of 200, 300, 400, 500, 600, 750 nm, pure SiO_2_ 0 nm (control) and flat TCP [blank control])	One-step self-assembly of PS nanospheres on SiO_2_ surface	[75]
Graphene	Nanoparticles of low-oxygen graphene	Commercially purchased	[76]
β-TCP	Roughness (from gelatin/reduced graphene oxide-magnesium-arginine hybrid contents of 0, 0.25 and 0.75% wt)	3D printing and freeze drying	[77]
GaN	Nanopores (etching voltage-nanopore size: 10 V: 20 nm; 15 V: 30 nm; 20 V: 80 nm; 25 V: 95 nm)	Electrochemical etching	[52]
Si	Nanopillars (critical dimensions of 40–200 nm)	Self-assembly block copolymer (spin-coating of PS-*b*-P2VP reverse micelle solutions)	[78]
C	Single and multi-walled nanotube-coated glass (nanostructures with height of 15–20 nm)	Coating and drying	[79]
Chitosan	Nanocomposite (CS scaffold with GO concentration of 2, 4 and 6% wt)	Direct blending, freezing and freeze-drying methods	[80]
Glass	Roughness (R*_q_* = 1, 100, 200 nm)	Reactive-ion etching	[53]

AuNW: Gold nanowire; BT NP: Barium titanate nanoparticle; C: Carbon; CaP: Calcium phosphate; CS: Chitosan; GaN: Gallium nitride; GO: Graphene oxide; GQD: Graphene quantum dot; HAP: Hydroxyapatite; HC: Honeycomb; PCL: Polycaprolactone; PDMS: Polydimethylsiloxane; PS: Polystyrene; PS-*b*-P2VP: Polystyrene-block-poly)2-vinylpyridine; PUA: Polyurethane acrylate; SBF: Simulated body fluid; Si: Silicone; SPION: Superparamagnetic iron oxide nanoparticle; TCP: Tricalcium phosphate; Ti: Titanium; TiNbTa: Ti-36Nb-Ta alloy.

**Figure 1.**
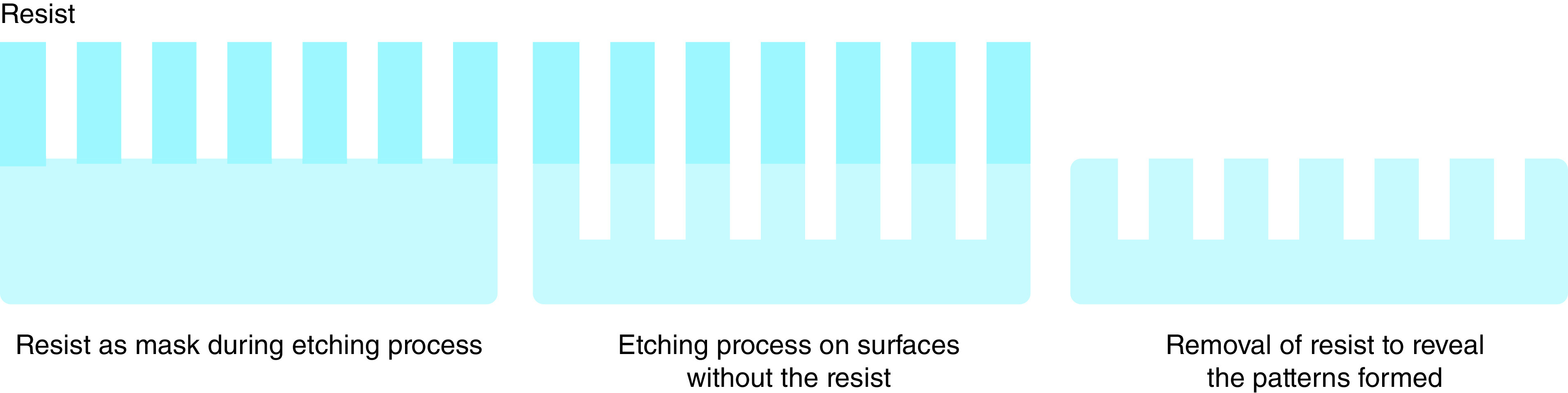
Example of lithographic process. Adapted with permission from [81] using Biorender.

Lithography is a fabrication method that prints a pattern onto a target surface through a thin layer called resist. After the resist pattern has been printed, a subtractive transfer is carried out, which etches the target surface but only on the parts without the resist so that it forms a pattern (Figure 1). Resist acts as a barrier during etching. Afterward, an additive transfer can also be carried out, which adds a layer of material to the opening that has been formed. There are many types of lithography, such as photolithography, x-ray lithography, electron beam lithography, nano-imprint lithography and others [81–83].

Etching is indeed part of the processes in lithography. It is the process of engraving on a surface and does not require printing patterns on the resist. For example, Han *et al.* used the electrochemical etching method to form nanopores on GaN films. The film was immersed in an electrolyte solution and then electrified with a certain voltage such that pores formed on the film randomly [52]. Several etching methods are wet etching, dry etching and reactive ion etching (RIE). Wet etching is a chemical etching process that uses chemical solutions; dry etching is an etching method using high-energy ions fired onto surfaces under airtight conditions; and RIE is a combination of chemical and physical etching via radiofrequency plasma [81].

The anodization method has been widely used in recent years. Patterns formed by this method are usually nanotubes using titanium and its alloys as the substrate material. Titanium has advantages as a biomaterial, specifically, that is light, strong, biocompatible and rust-resistant. However, titanium's Young's modulus is often not enough to be used as a biomaterial, and therefore titanium is usually replaced with its alloy Ti6Al4V [84,85]. Anodization is the most popular method used for titanium material. This method is also relatively easy to do and does not require sophisticated tools. As the diameter of the resulting nanotubes can be customized by adjusting the applied voltage, the cost is not excessive. Tong *et al.* proved that western blot results in several osteogenic marker genes have higher relative mRNA expression on surfaces with nanotubes than those in flat surface controls [55].

Based on the three types of methods previously discussed, the most widely used type of nanopattern in the last 2 years is nanotubes produced through anodization. This method is commonly used for titanium material, which does have good performance in bone tissue engineering both *in vitro* and *in vivo* [86]. Among the three methods discussed, anodization is a relatively simple and easy to perform and regulate because it uses the principle of electrochemical oxidation with voltage variations. Although this method generally forms nanotubes, it can also be combined with other methods to form other types of nanopatterns. One example is nanopores by electrochemical nanopattern formation (ENF) which consists of anodization, sonication and chemical etching [64]. Considering anodization's simplicity, flexibility regarding customization, suitability of the material, and osteogenic differentiation ability, it has the potential for further developments in bone tissue engineering.

## Role of nanopatterns in osteogenic differentiation induction

Nanopatterns were developed in the field of stem cell culture mainly to direct cell differentiation to the cell line that suits specific needs. Generally, cell differentiation is induced by chemical compounds such as growth factors. However, the use of chemical compounds for induction has its limitations. Growth factors need to be used in large doses to achieve the appropriate phenotype. Consequently, the costs can be great. In addition, maintenance of hMSCs *in vivo* after they are administered to patients is also very difficult [87].

Research on nanopatterns is driven by the knowledge that small changes in topography can have a significant influence in directing stem cell development [69]. For example, Kim *et al.* used several types of nanopatterns functionalized by BMP-2 (bone morphogenetic protein-2). They investigated how stem cells respond when grown on functionalized (compared with nonfunctionalized) nanopatterns, and with the addition (compared with lack) of osteogenic induction. qRT-PCR results of osteogenic markers expression (Col I, *RUNX2*, OPN) showed that surfaces with nanopatterns could upregulate osteogenic markers expression when compared with flat surfaces and further increased with BMP-2 functionalization [27].

Besides the presence of nanopatterns, the shapes and sizes of nanopatterns can also affect differentiation. Kim *et al.* showed that different patterns further increase different osteogenic markers expression. Col I and *RUNX2* were expressed mainly in hexagonal dot patterns in substrates without functionalization and without induction medium. On substrates with induction medium, Col I expression was enhanced on substrates with BMP-2 functionalization and was more visible on groove-patterned substrates than dot-patterned substrates [27]. Li *et al.* demonstrated that differences in nanotubes diameter can affect differentiation. Fluorescence staining results showed that the levels of osteocalcin (OC), osteopontin (OP), and xylenol orange (calcification) were higher on substrates with 39 nm (20 V) diameter than 83 nm (40 V) [60].

Although nanopatterns can induce osteogenic differentiation, studies have shown that nanopatterns alone are not effective enough compared with using a combination of osteogenic induction medium with nanopattern. You *et al.* showed that ALP and Cbfa1 were expressed both in nanopattern with osteogenic medium and with growth medium only. However, OP and OC were significantly lower in nanopatterns with growth medium compared with those using the osteogenic medium. These results show that early-stage differentiation can be initiated using nanopatterns and growth medium only because there are no significant differences with when an osteogenic medium is used. However, for later-stage osteogenic differentiation, stronger induction from the osteogenic medium is required [50].

Further, Watari *et al.* conducted a study that was slightly similar to You *et al*. They used nanopatterns without osteogenic medium and nanopatterns with an added osteogenic medium. Nanopatterns with pitch sizes of 400 nm can significantly increase the expression of *RUNX2*, BGLAP and calcification compared with controls with or without an added osteogenic medium. The combination of nanopatterns with the osteogenic medium can provide additional effects that may be beneficial for people with serious conditions. Its effects as a biophysical signal on osteogenic differentiation can also last up to 2 weeks, whereas those from the biochemical medium usually last only 8 days or fewer [88]. The increase in expression on nanopatterns compared with control was higher in the non-osteogenic medium compared with the osteogenic medium. This result was observed on *RUNX2* on day 3, BGLAP on day 10, and calcification on day 14. Thus, although nanopatterns cannot completely replace osteogenic medium, nanopatterns themselves can increase the expression of osteogenic markers compared with the control. Additional osteogenic medium is then beneficial to further increase the expression, if more is needed. Further research is needed to confirm whether using nanopattern alone for early differentiation and using induction medium for late differentiation generates consistent results, how this approach compares to using nanopatterns alone or using osteogenic medium from the beginning, and also what is the underlying mechanism.

Although nanopatterns can enhance osteogenic differentiation, as shown through osteogenic markers expression, the process still requires the addition of osteogenic mediums. Therefore, when using nanopatterns to achieve a certain level of differentiation, the required biochemical compounds for the osteogenic medium is reduced. Qian *et al.* used substrates with a roughness level of 1 and 200 nm with five types of mediums, namely control medium, complete osteogenic induction medium, induction medium without dexamethasone, induction medium without ascorbic acid, and induction medium without β-glycerophosphate. His research showed that the level of ALP expression was higher in the induction medium without dexamethasone compared with control, medium without ascorbic acid, and medium without β-glycerophosphate. Expression of *RUNX2*, ALP, and OPN on nanorough along with induction medium without dexamethasone was higher compared with the flat surface with complete osteogenic induction medium [53]. Another study, by Thiagarajan *et al.*, stated that dexamethasone, which is commonly used for osteogenic differentiation, can trigger pleiotropic effects, which means it can cause adverse effects on the body. Two other compounds commonly used in inducing osteogenic differentiation, ascorbic acid and β-glycerophosphate, function to promote collagen production and supply inorganic phosphate for mineralization *in vitro*. Because these two compounds are naturally available *in vivo*, inducing agents aren't necessarily needed [89]. If nanopatterns can indeed replace dexamethasone's role, the number of induction compounds needed and also the risk of unwanted effects are also reduced. Nanopatterns with the addition of ascorbic acid and β-glycerophosphate alone may be sufficient during *in vitro* conditions, and during *in vivo*, induction effects can be maintained by the presence of nanopatterns.

These studies prove that nanopatterns can enhance osteogenic differentiation either alone or in combination with osteogenic media. The findings indicate that nanopatterns can also reduce the costs required for biochemical compounds, either by reducing their use or by increasing the expression of osteogenic markers, and hence make their use more efficient. Higher marker expressions on surfaces with nanopatterns indicate that stem cells' development into bone cells occurs faster than on surfaces without nanopatterns. Further, mechanical signals from nanotopography have the advantage of being more stable, controlled, and more quickly transmitted to the nucleus than chemical signals [90]. With the right size, nanopatterns can also form stable focal adhesion [91]. Focal adhesion stability can keep cytoskeletal tension stable in order for signals from nanopatterns to reach the nucleus properly. Initial cell attachment to the surface can affect osteogenic differentiation and the long-term stability of scaffolds after transplantation. Rapid and stable osseointegration, which is recognized through the cell's osteogenic differentiation rate, can reduce the risks of failure after implantation [65]. Thus, nanopatterns can be a very important first step in directing osteogenic differentiation and maintaining the stability of the differentiation process.

One of the important aspects to consider in tissue engineering implementation, besides biology and security, is the cost. As discussed, nanopatterns are generally able to show higher expression of osteogenic markers compared with controls. This expression is even higher when nanopatterns are combined with an osteogenic induction medium. Therefore, nanopatterns can accelerate the process of osteogenic differentiation and also reduce the use of growth factors. Nanopatterns can also provide more stable signaling compared with biochemical compounds. With these considerations, nanopatterns are economical and worth further development. Research in this area is still very wide open, including further comprehensive research on the financial aspect to see whether nanopatterns can reduce the use of growth factors, and thus be not only safer but also even more economical.

## Nanopatterns potential in bone tissue engineering

The use of nanopatterns is currently still in the research stage. However, the ultimate goal of nanopattern research is for application, specifically in bone treatment by either injecting differentiated stem cells or transplanting them together with the scaffolds into the body. If transplanted, the nanopatterns must already be embedded in the scaffold, and the scaffold must be biocompatible in order to be transplanted into the body and to facilitate bone cells growth. In general, scaffolds for bone tissue engineering must meet several criteria. Biological criteria of scaffolds include biocompatibility, nontoxicity and biodegradability. In addition to biological criteria, there are also mechanical criteria. Scaffolds must be mechanically designed according to natural bone tissues to prevent complications. Finally, there are structural criteria, which include having certain levels of porosity to improve osseointegration and also being harnessed with nanotopography [92].

Nanotopographic structures of the scaffold play an important role in osteoinduction and also osteointegration (ability to integrate with bone). Nanopatterns provide biophysical signals and are transmitted through mechanotransduction into the nucleus with the help of integrins and protein complexes in the cell. Integrin will bind to proteins such as FAK, talin, vinculin and paxillin which will indirectly be connected to actin cytoskeletal components and subsequently will affect gene expression via signaling cascades. However, under conditions with nanopatterns, the FA formed is larger so that F-actin will undergo crosslinking to form stress fibers. Cell shape becomes tenser so that it can facilitate osteogenesis better [34]. Thus, biophysical signals from nanopatterns can regulate the expression of genes related to proliferation as well as differentiation of MSCs into osteoblasts.

**Figure 2.**
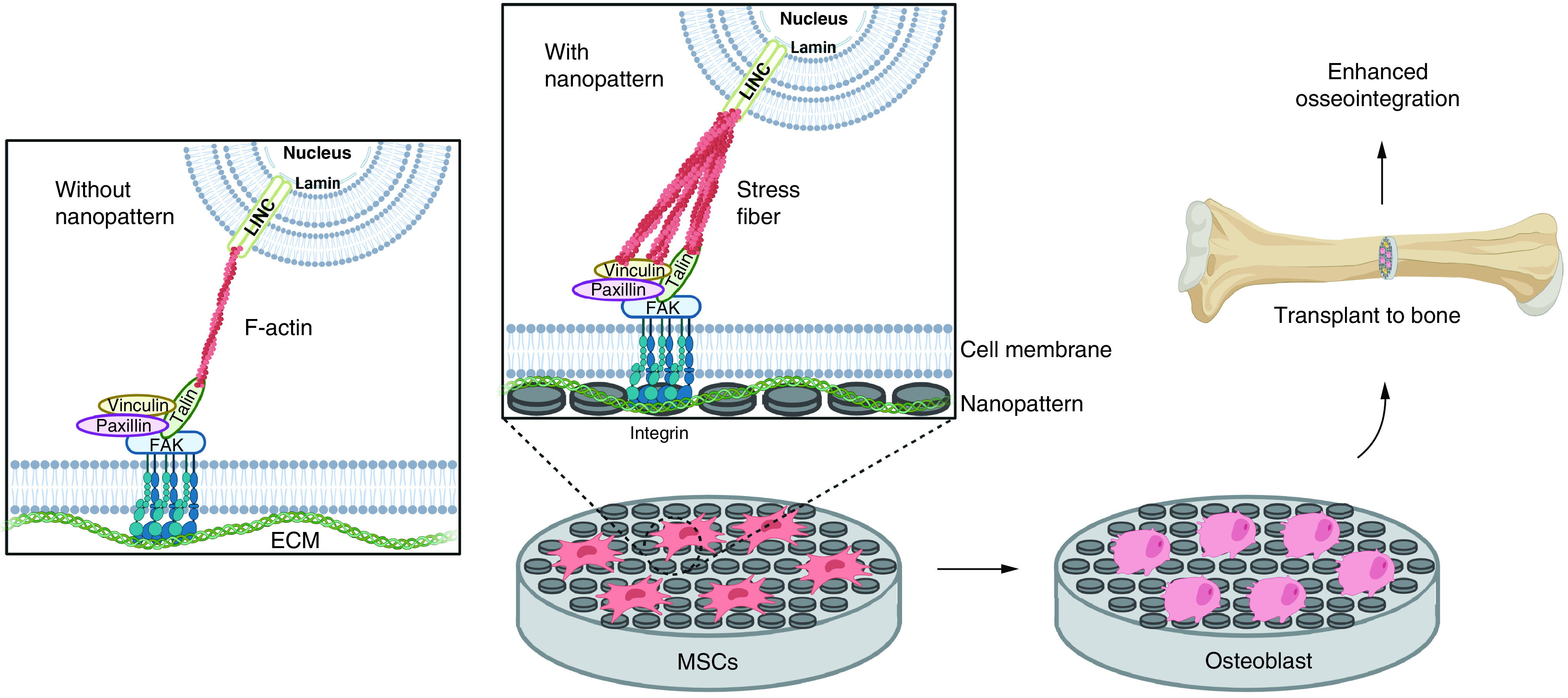
A simple diagram of the mechanism of nanopatterns mechanotransduction pathway on scaffolds and their application in transplantation. Created using Biorender. ECM: Extracellular matrix; MSCs: Mesenchymal stem cells.

Scaffolds can be transplanted into damaged bones when cells have reached the osteoblast stage. Osteoblasts can interact with other osteoblasts as well as with osteocytes. Osteoblasts can secrete matrixes that are beneficial for the mineralization process of osteoblasts themselves. Then, osteocytes present in bones can provide paracrine stimulation of osteoblasts to form more osteocytes and also inhibit osteoclast formation [93]. A functional scaffold that has a good degree of osseointegration will have a density similar to the natural bones with which it is integrated. Nanopatterns can affect the conformation of adsorbed proteins and increase attachment by integrins and ultimately promote osseointegration (Figure 2) [93,94]. Of course, the entire process requires support from plenty of research because the mechanisms involved are very complex.

Nanopatterns on scaffolds will be able to maintain osteogenic induction effects after being transplanted because nanopatterns are already part of the scaffold, in contrast to biochemical compounds whose effects can decrease if not continuously supplemented. Notably, after transplantation, administrations of induction compounds pose some risks to the person. Negative effects that may be caused by biochemical compounds, such as the pleiotropic effect of dexamethasone, can be avoided by utilizing nanopatterns [89]. In this case, nanopatterns have benefits compared with adding growth factors.

Materials for scaffolds, as previously discussed, are very important and have certain criteria. Different types of materials have been used for nanopattern research in inducing osteogenic differentiation, such as barium titanate ceramics [28], PDMS [74] and PS [75] polymers, graphene [76], silicones [78], carbon nanotubes [79], chitosan [95] and many others. Akhavan *et al. *showed one of the first works relating to the effect of surface nanotopography from graphene (surface coated by graphene nanoribbons in the form of nanogrids) on MSC's proliferation and differentiation [96]. Recently, graphene has been used in combination with nanostructures to create substrates that can enhance osteogenesis. Therefore, graphene are also highly promising because of its biocompatibility and cost–effectiveness [97,98]. However, titanium and its alloys are the most commonly used according to literatures [57–60,62–66,68]. Titanium has been mentioned as an advantageous material and can be fabricated into nanotubes by anodizing. However, the main disadvantage of using titanium as a material for scaffolds is that titanium is a non-biodegradable metal. Therefore, during the process of bone healing, a further operation is needed to remove the scaffold because there is the possibility of problems emerging in the long term. To address this issue, research on biodegradable metals has been carried out, for example magnesium alloys. However, the degradation of magnesium implant material in the body occurs through corrosion, raising concerns about its effects on the body [92]. Other biodegradable materials have also been widely used as scaffolds in nanopattern research. Some examples are polymers such as PCL [70–73], bioceramics such as calcium phosphate (CP) and β-TCP [69,77], and natural polymers such as chitosan [80]. Cellulose nanofibers with specific morphologies have also been used and may serve as a greener option [99]. It is known as a very effective, useful and biocompatible material for osteogenic differentiation. Composite nanofiber scaffolds from nano-sized demineralized bone powders (DBP) and biodegradable poly(L-lactide) (PLA) have also been used for *in vivo* bone formation. The osteoconductive effect of PLA/DBP scaffolds showed greater results compared with PLA scaffolds and the composite scaffolds *in vitro* [100]. Further research on suitable scaffold materials is still needed, especially materials that can facilitate the differentiation process toward osteogenicity.

## Conclusion & future perspective

Nanopattern as a method to induce osteogenic differentiation of MSCs has been widely studied. Various methods to produce nanopatterns have been and are being developed, and their development is highly dependent on the advancement of nanotechnology. Anodization has been researched widely in the last 2 years and has the potential to be further developed because it is quite simple and flexible regarding being varied. This method is usually used to create nanotube patterns that have been shown to increase osteogenic markers expression, which also indicates a faster differentiation process when compared with a flat surface.

However, the use of nanopatterns does not mean that growth factors do not need to be used. Combinations of nanopattern and growth factors can increase the effectiveness of osteogenic differentiation compared with nanopatterns alone. Nanopatterns can reduce the use of growth factors, which is beneficial for reducing costs and risks of side effects that can be caused by growth factors. In addition, mechanical signals provided by nanopatterns can provide a more stable, controlled, and faster signal that is transmitted to the nucleus.

In tissue engineering, nanopatterns can be applied in scaffolds such that their induced effects can persist even *in vivo*. The material used for scaffolds is also important. Titanium is one of the widely materials for bone implants because of its suitable characteristics. However, the use of metal has drawbacks because it is not biodegradable. Therefore, the development of biomaterials for nanopatterned scaffolds is wide open for exploration in the future for bone tissue engineering applications.

This short review has discussed a little about the nanopattern fabrication method and its role in inducing osteogenic differentiation, especially for tissue engineering. It provides a perspective regarding the potential use of nanopattern and recent advances in nanopattern function toward osteogenic differentiation of MSC for bone tissue engineering. Nanopatterns have great potential, especially in bone treatment, that further research and development may shed light on. It provides a perspective regarding the potential use of nanopattern and recent advances in nanopattern function toward osteogenic differentiation of MSC for bone tissue engineering.

**Executive summary**

- Nanopatterns have great potential in tissue engineering because they can induce mesenchymal stem cell differentiation in scaffolds via biophysical signals.
- Regulation of mesenchymal stem cell behavior by biophysical signals occurs through mechanotransduction.
- Anodization can be a method with great potential for further development in bone tissue engineering.
- Nanopatterns can increase osteogenic markers expression and reduce costs of production and maintenance.
- A combination of nanopatterns and growth factors can further increase the effectivity of osteogenic differentiation induction; hence biochemical compounds are not required in large quantities.
- Nanopatterned scaffolds made of suitable materials can improve osseointegration in bone tissue treatment.
